# Habitual Sleep Duration and Risk of Childhood Obesity: Systematic Review and Dose-response Meta-analysis of Prospective Cohort Studies

**DOI:** 10.1038/srep16160

**Published:** 2015-11-05

**Authors:** Huijuan Ruan, Pengcheng Xun, Wei Cai, Ka He, Qingya Tang

**Affiliations:** 1 Department of Clinical Nutrition, Xin Hua Hospital, School of Medicine, Shanghai Jiao Tong University, Shanghai, China; 2 Department of Epidemiology and Biostatistics, School of Public Health-Bloomington, Indiana University, Bloomington, IN, USA; 3 Shanghai Key Laboratory of Pediatric Gastroenterology and Nutrition, Shanghai, China; Shanghai Institute of Pediatric Research, Shanghai, China; 4 Department of Pediatric Surgery, Xin Hua Hospital, School of Medicine, Shanghai Jiao Tong University, Shanghai, China

## Abstract

A meta-analysis of cross-sectional studies found that shorter-time sleep was correlated with increased risk of obesity in children. However, findings from prospective cohort studies were inconsistent. PubMed and other data resources were searched through May 2015. Twenty-five eligible studies were identified including 56,584 children and adolescents with an average 3.4-year follow-up. Compared with children having the longest sleep duration (~12.2 hours), kids with the shortest sleep duration (~10.0 hours) were 76% more likely to be overweight/obese (pooled odds ratio [OR]: 1.76; 95% confidence interval [CI]: 1.39, 2.23); and had relatively larger annual BMI gain (pooled *β* coefficient: 0.13; 95% CI: 0.01, 0.25 kg/m^2^). With every 1 hour/day increment in sleep duration, the risk of overweight/obesity was reduced by 21% (OR: 0.79; 95% CI: 0.70, 0.89); and the annual BMI gain declined by 0.05 kg/m^2^ (*β* = −0.05; 95% CI: −0.09, −0.01). The observed associations were not appreciably modified by region, baseline age or the length of follow-up. Accumulated literature indicates a modest inverse association between sleep duration and the risk of childhood overweight/obesity. Further research is needed to determine the age and gender specified optimal hours of sleep and ideal sleep pattern with respect to obesity prevention in children.

A number of epidemiological studies have suggested that childhood obesity is an independent risk factor of chronic diseases later in life^1^,^2^,^3^,^4^. Thus, identifying modifiable risk factors of obesity in childhood is of great public health significance.

Sleep duration, a modifiable factor, was suggested to play an important role in the development of childhood obesity. A meta-analysis that summarized data from 12 cross-sectional studies found that shorter-time sleep was correlated with an increased risk of obesity in children^5^. Findings from prospective cohort studies were inconsistent. One recent systematic review assessed the longitudinal studies in children and adolescents and concluded that the majority of the studies supported an inverse association between sleep duration and the risk of childhood overweight/obesity^6^. However, that review did not quantitatively estimate the overall association, particularly the dose-response relationship. Therefore, we aimed to examine the longitudinal association between habitual sleep duration and the risk of childhood overweight/obesity as well as the related anthropometric parameters by quantitatively summarizing data from prospective cohort studies.

## Methods

The present meta-analysis was conducted following the guidelines of the Meta-analysis Of Observational Studies in Epidemiology (MOOSE)^7^. We systematically reviewed the literature in PubMed and EMBASE through May 2015 to identify prospective cohort studies published in English on the association between sleep duration and the risk of overweight/obesity in children and adolescents. We used the key words “*sleep*” and “*obesity*”, “*adiposity*’, “*body mass index*”, “*body weight*”, “*waist circumference*”, or “*skinfold thickness*”, and “*follow-up studies*”, “*longitudinal studies*”, or “*prospective studies*” and “*children*” or “*adolescents*”. In addition, we searched Google Scholar and manually reviewed the reference lists from the relevant articles.

Eligible studies were prospective cohort studies conducted in children and/or adolescents, and reported results on the association between sleep duration and the risk of overweight/obesity and/or anthropometric measures. The primary outcomes included the risk of overweight/obesity and annual body mass index (BMI) gain. The secondary outcomes included BMI, BMI z-score [created from BMI (kg/m^2^) according to the 2000 Centers for Disease Control (CDC) growth reference], weight, waist circumference (WC), percent body fat (PBF), fat free mass index (FFMI), fat mass (FM), fat mass index (FMI), and sum of skin-folds (SSF). For multiple publications using data from the same cohort, the one with the longest follow-up period or the largest sample size was selected for this meta-analysis.

### Data extraction

Two authors reviewed the literature independently and extracted the information for the meta-analysis following a formal protocol written in advance that clearly stated the objectives, the hypotheses to be tested, the subgroups of interest, and the proposed methods and criteria for identifying and selecting relevant studies and extracting and analyzing information^8^. Data extraction covered 1) general information of the study: first author’s name, study name (if applicable), year of publication, and country where the study was conducted; 2) characteristics of study population: age, total number of participants, and percent of boys; 3) assessment and categorization of exposure; 4) ascertainment of outcome; 5) covariates adjusted in the analysis; and 6) measures of the association, e.g., odds ratio (ORs) and *β* coefficients and corresponding 95% confidence intervals (CIs). Discrepancies on literature review and data extraction were resolved by group discussion.

### Statistical Analysis

According to the recommendation of the World Health Organization (WHO), overweight was defined as an age and gender specific BMI between the 85^th^ and 95^th^ percentile and obesity was defined as a BMI above the 95^th^ percentile^9^. The average follow-up time was calculated as the sum of person-years divided by the total number of participants.

To estimate the overall association between sleep duration and risk of overweight/obesity, we used the inverse of variance as the weight to calculate the pooled ORs and 95% CIs comparing the shortest to the longest category of sleep duration. Standard errors (SEs) were derived from the fully adjusted ORs and 95% CIs in the primary studies, which were transformed to natural logarithms (ln). To estimate the association between sleep duration and the continuous outcomes (e.g. annual BMI gain), we pooled the *β* regression coefficients weighted by the inverse of their variances considering that both exposure (i.e. sleep duration) and outcomes (e. g. annual BMI gain) were measured similarly in the primary studies^10^. If the information on linear association was not available in the primary study, it would be derived from generalized least-squares for trend test if the number of data points was ≥3^11^, or calculated directly under a linear assumption if the number of data points was <3. If the extreme sleep duration category was open-ended (e.g. ≥12 hours/day), its lower/upper limit was estimated by assuming the range equivalent to its adjacent close-ended category.

To assess heterogeneity among the original studies, we inspected forest plots and conducted a Cochran’s *Q* test with a *P* ≤ 0.10 considered as significant heterogeneity. We also computed the *I*^2^ statistic to measure the magnitude of heterogeneity. The low, moderate, and high levels of heterogeneity were defined as <30%, 30–50%, and >50%, respectively. Sources of heterogeneity were explored using meta-regression and subgroup analyses with pre-defined factors including age (<3, 3- < 5, ≥5 years), gender, follow-up time (above or below median) and study region (USA *vs*. non-USA).

Small-study effects or publication bias was assessed by funnel plot asymmetry followed by Egger’s regression asymmetry test (when the number of studies was ≥3) or Begg’s adjusted rank correlation test (when the number of studies was <3). The Duval and Tweedie nonparametric “trim and fill” method was used to adjust for publication bias, if needed^12^.

Results from a random-effects model were presented as our main findings because we found that there were moderate/high heterogeneities in most of the pooled analyses and publication bias existed in some analyses^13^. Sensitivity analyses were performed to evaluate the robustness of the findings. Specifically, we determined the effects of a single study on the pooled results by removing one study at a time in the meta-analysis. Also, we explored the possible changes if replacing a random-effects model with a fixed-effects model.

All analyses were performed using STATA statistical software (Version 13.0; STATA Corporation LP, College Station, Texas, USA). A two-sided *P* value ≤0.05 was considered significant if not specified.

## Results

### Literature search

**Figure 1:**
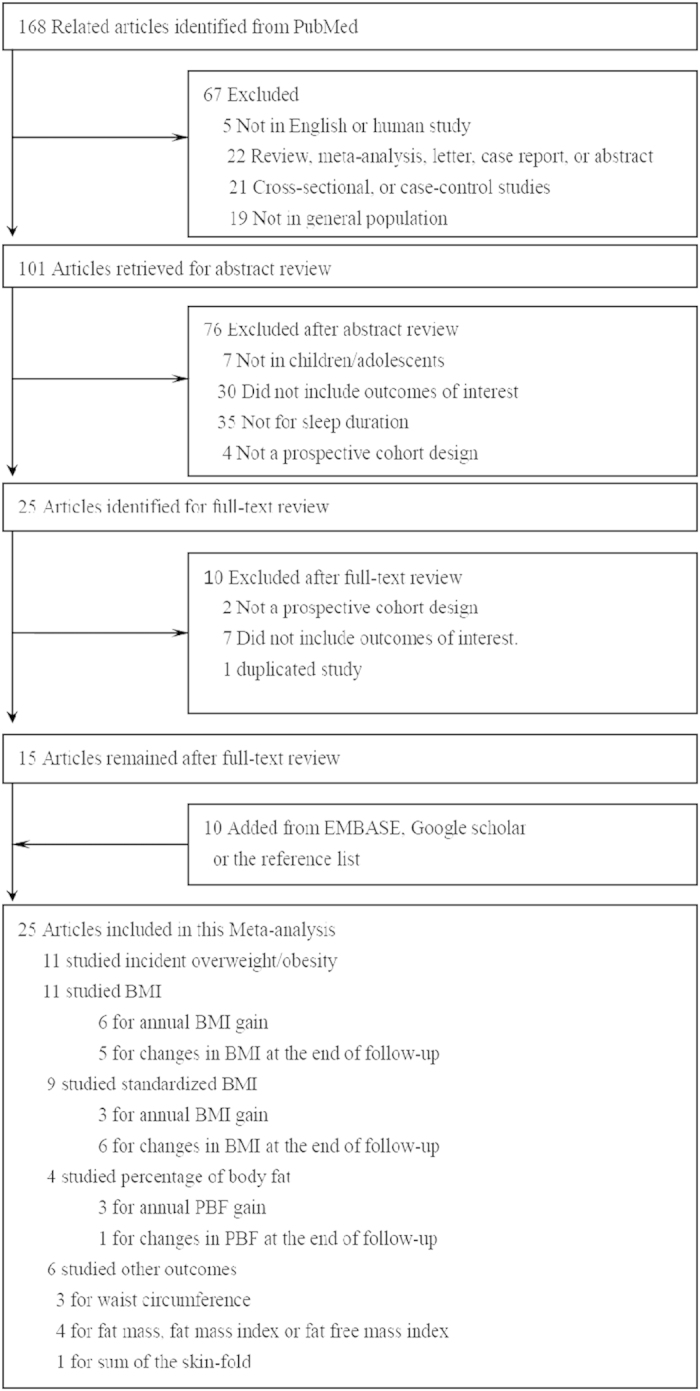
Flowchart of study screening and selection.

As shown in Figure 1, 168 relevant articles were retrieved from PubMed. Of them, 145 were excluded for one of the following reasons: 1) not in English (n = 5); 2) not original studies (e.g., letter to editor) (n = 22); 3) not prospective cohort studies (n = 26); 4) not in children/adolescents (n = 26); 5) not on sleep duration or no outcome of interest (n = 72); 6) no sufficient information on measures of the association of interest (n = 1). In addition, 10 studies were identified from EMBASE, Google Scholar, or the reference lists of the relevant articles, for a total of 25 eligible studies in the meta-analysis^14^,^15^,^16^,^17^,^18^,^19^,^20^,^21^,^22^,^23^,^24^,^25^,^26^,^27^,^28^,^29^,^30^,^31^,^32^,^33^,^34^,^35^,^36^,^37^,^38^.

### Characteristics of included studies

Table 1 presents characteristics of the 25 included studies. Eleven studies were conducted in the USA^15^,^16^,^17^,^19^,^20^,^25^,^29^,^31^,^33^,^37^,^38^, four in Australia^23^,^28^,^32^,^35^, two in Canada^18^,^24^, two in Denmark^30^,^34^, and one in each of the following countries: UK^14^, Germany^22^, New Zealand^21^, Portugal^26^, Korea^27^, and Belgium^36^. Some studies reported results separately based on two^19^,^23^,^27^,^30^ or three different age groups^38^, or separately by genders^29^. We treated them as independent cohorts. In the final dataset, 56,584 children and adolescents from 25 studies (32 independent cohorts), with an average 3.4-year follow-up, were included.

Information on sleep duration and the risk of overweight/obesity were available in 11 studies (14 cohorts)^14^,^15^,^16^,^17^,^18^,^19^,^20^,^21^,^24^,^25^,^38^, including 31,185 participants and 4,473 cases with a follow-up range from 21 months to 5 years. Data on sleep duration and annual BMI gain were available in six studies (8 cohorts)^22^,^25^,^27^,^28^,^31^,^33^ including 5,341 participants with a follow-up range from 2 to 5 years.

### Sleep duration and risk of overweight/obesity

**Figure 2:**
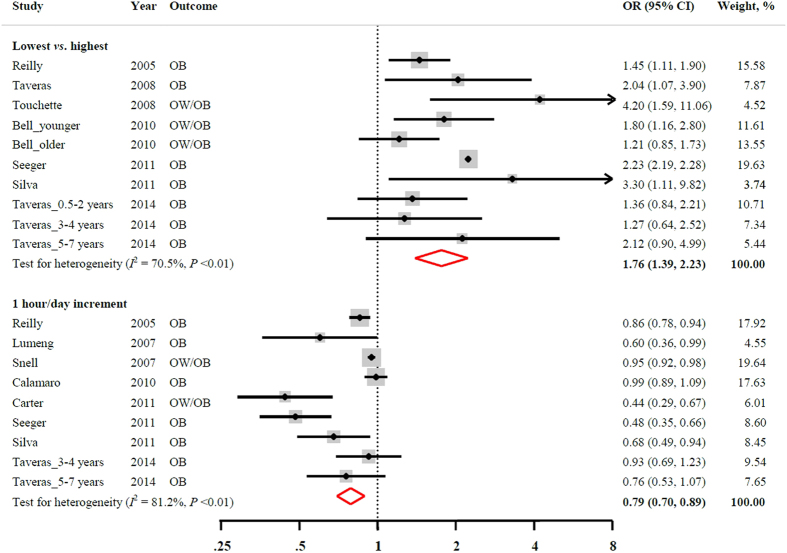
Multivariable-adjusted OR and 95% CI of overweight/obesity in relation to sleep duration. The overall estimates are obtained by using a random-effects model. The dots indicate the adjusted ORs comparing the lowest to the highest levels of sleep duration or every 1 hour/day increment in sleep duration. The size of the shaded square is proportional to the weight of each study. The horizontal lines represent 95% CIs. The diamond indicates the pooled OR. CI: confidence interval; OB: obesity; OR: odds ratio; OW: overweight.

Figure 2 shows the association between sleep duration and the risk of overweight/obesity. By combining data from seven studies (10 independent cohorts), the pooled OR (95% CIs) was 1.76 (95% CI: 1.39, 2.23) for participants in the shortest sleep duration group as compared with those who were in the longest sleep duration group. The heterogeneity was high among studies (*I*^2^ = 70.5%, *P* < 0.01). No strong evidence of publication bias was observed (Egger’s test, *P* = 0.10).

For the dose-response relationship, after pooling available data from 8 studies (9 independent cohorts), the risk of overweight/obesity was 21% lower with every 1 hour/day sleep duration increment (combined OR: 0.79; 95% CI: 0.70, 0.89). A high heterogeneity among studies was observed (*I*^2^ = 81.2%, *P* < 0.01). Because Egger’s test indicated a significant publication bias (*P* = 0.01), the “trim and fill” method was used to adjust for the publication bias. With this adjustment, the pooled linear association was somewhat attenuated and became statistically non-significant (pooled OR: 0.92; 95% CI: 0.81, 1.05).

### Sleep duration and annual BMI gain

**Figure 3:**
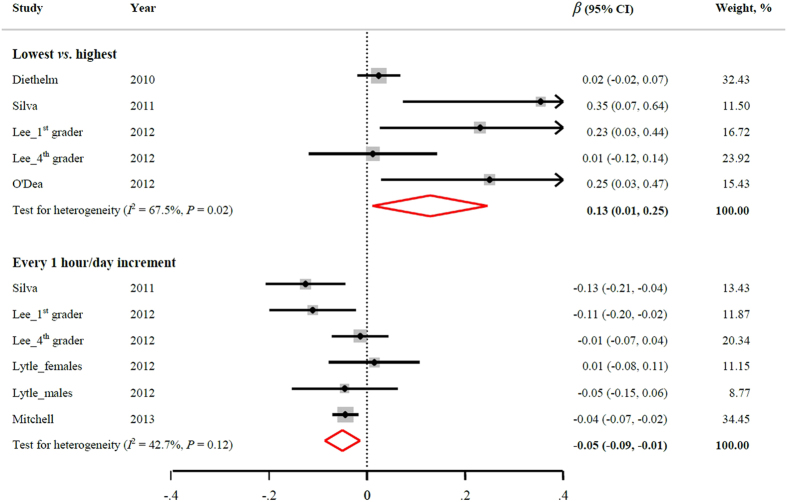
Multivariable-adjusted *β* coefficient and 95% CI of annual BMI gain in relation to sleep duration. The overall estimates are obtained by using a random-effects model. The dots indicate the adjusted *β* coefficients comparing the lowest to the highest levels of sleep duration or every 1 hour/day increment in sleep duration. The size of the shaded square is proportional to the weight of each study. The horizontal lines represent 95% CIs. The diamond indicates the pooled *β* coefficient. BMI: body mass index; CI: confidence interval.

Figure 3 demonstrates the relation between sleep duration and annual BMI gain. Four studies (5 independent cohorts) provided data on the multivariable-adjusted *β* coefficient (95% CIs) comparing children with the shortest to the longest sleep duration. The pooled results indicated that participants with the shortest sleep duration had significantly more annual BMI gain (*β* coefficient: 0.13; 95% CI: 0.01, 0.25). High heterogeneity among studies existed (*I*^2^ = 67.5%, *P* = 0.02). The test for publication bias approached significance (Egger’s test, *P* = 0.054).

For the dose-response relationship, 4 studies (6 independent cohorts) had data available. Every 1 hour/day sleep duration increment would decrease annual BMI gain by 0.05 kg/m^2^ (*β* = −0.05; 95% CI: −0.09, −0.01). A moderate heterogeneity was observed (*I*^2^ = 42.7, *P* = 0.12). There was no evidence indicating publication bias (Egger’s test, *P* = 0.70).

### Subgroup and sensitivity analysis

According to the available data, we examined potential effect modification by study region (USA *vs*. Non-USA), age (<3, 3 < 5, ≥5 year), or follow-up duration (above or below the median). None of these factors materially modified the observed associations (Table 2). A few studies^26^,^29^,^31^ examined gender difference between sleep duration and BMI z-score or PBF. Although the number of studies is not sufficient for us to pool the results, none of these studies found a significant gender difference.

In addition, the results were not appreciably changed when replacing random-effects models with fixed-effects models (data not shown). No single study substantially influenced the pooled results on sleep duration and the risk of overweight/obesity when omitting one study each time in the meta-analysis (Supplemental Table). However, the association between sleep duration and annual BMI gain was substantially attenuated when removing one of three independent cohorts (Silva, 2011^25^, Lee, 2012^27^, or O’Dea, 2012^28^) at a time (Supplemental Table).

### Sleep duration and secondary outcomes

Table 3 shows the association between sleep duration and secondary outcomes. Sleep duration was inversely associated with BMI, BMI z-score and WC, and positively related to FMI.

## Discussion

This meta-analysis of prospective cohort studies provides accumulated evidence supporting the hypothesis that sleep duration in children and/or adolescents is inversely associated with the risk of overweight/obesity in a dose-response manner. This inverse association is not appreciably modified by study region, baseline age or the length of follow-up.

### Strengths and limitations

This meta-analysis was based on up-to-date literature with the largest synthesis of prospective cohort studies from various populations. The overall sample size was large (*n* = 56,584) and the average follow-up period was relatively long (~3.4 years) among children and adolescents. Data for the pooled analysis were derived from fully adjusted models in the primary studies, which should reduce the likelihood of confounding. Also, our conclusions were strengthened by the consistent findings in the binary associations (the shortest *vs*. the longest sleep duration), the dose-response relationships, and the results of other related anthropometric measures such as annual BMI gain and WC.

A few limitations of this meta–analysis should also be considered. First, because sleep duration was parent- or self-reported, misclassification was possible, though it was likely to be non-differential and may attenuate the observed associations. Second, incident cases of overweight/obesity in the primary studies could not be determined. However, this was a common methodological issue in the studies of childhood obesity. Third, sleep durations were classified differently across the original studies, especially the reference groups were somewhat different. This inconsistency could affect our pooled results. Fourth, the findings could be biased by residual confounding or unmeasured confounders in the primary studies. Fifth, results from some sub-group analyses might not be very robust because of the high heterogeneity across the primary studies. Finally, publication bias was found when assessing linear association between sleep duration and the risk of overweight/obesity. However, the conclusion generally remained after controlling for the publication bias using Duval and Tweedie’s “trim and fill” method.

### Comparison with previous reviews or meta-analyses

A few previous narrative or systematic reviews found an inverse association between sleep duration and risk of obesity or weight gains among children and adolescents^6^,^39^,^40^,^41^,^42^. However, these reviews did not conduct any quantitative analysis. A meta-analysis published in 2008 found that children or adolescents with shorter sleep duration had a 58% higher risk of overweight/obesity^43^. Of note, nine out of eleven studies included in that meta-analysis were cross-sectional, and only 2 were longitudinal studies. Similar results were reported in the same year in another meta-analysis, which included 12 cross-sectional studies^5^. A recently published meta-analysis focused on longitudinal studies in children and adolescents^44^, but this analysis did not examine the dose-response relationship and other related anthropometric parameters. Thus, the present study certainly contributes important additional information to the literature.

### Potential mechanisms

The etiology of obesity is multifactorial, in which genetic^45^, metabolic^46^, environmental^47^, behavioral^48^, and social or cultural factors^49^,^50^ are major contributors. Although the exact mechanism remains mysterious, there are several possible pathways that could explain the inverse association between sleep duration and the risk of obesity/overweight. First, sleep has a beneficial effect on the sympathetic nervous system and hypothalamic hormones^6^. For example, sleep deprivation is associated with low levels of leptin and high levels of ghrelin, which suggests an important role in appetite regulation^51^,^52^. Sleep restriction is also associated with an increase in cortisol levels, which promotes increased food intake and the accumulation of visceral fat^6^. Second, sleep restriction or deprivation may lead to reduced energy expenditure by reducing thyroid-stimulating hormone (TSH), and therefore reducing basal metabolic rate, or by breaking the balance of neural circuits that maintain body weight. Third, sleep restriction can contribute to obesity by promoting waking behavior that causes weight gain. For instance, increased waking hours may provide more opportunities for an individual to eat more energy dense foods^53^,^54^. Also, lack of sleep is associated with sedentary activities such as television watching that might lead to weight gain due to increased snacking^35^. In addition, people with sleep restriction often experience fatigue and sleepiness throughout the day, which may cause a decrease in physical activity^53^. It is also possible that people with short sleep duration might drink/eat more high-energy drinks/foods to conquer fatigue^53^.

### Summary

In conclusion, this meta-analysis of prospective cohort studies, based on up-to-date literature, provides moderate evidence that sleep duration may be inversely and longitudinally associated with the risk of overweight/obesity in children and adolescents. To prevent childhood obesity and, thereafter, chronic diseases, a certain amount of sleep time for children and adolescents should be recommended. Since a randomized clinical trial on sleep duration may not be feasible due to ethical and practical considerations, further prospective cohort studies with longer follow-up periods, valid objective measures of sleep duration and dynamic sleep quality, as well as multiple measures of body composition, are warranted to further explore age and gender specified optimal sleep duration, and ideal sleep pattern (e.g., sleep timing and chronotype) in terms of weight maintenance.

## Additional Information

**How to cite this article**: Ruan, H. *et al.* Habitual Sleep Duration and Risk of Childhood Obesity: Systematic Review and Dose-response Meta-analysis of Prospective Cohort Studies. *Sci. Rep.* **5**, 16160; doi: 10.1038/srep16160 (2015).

## Supplementary Material

Supplementary Information

## Figures and Tables

**Table 1:** Characteristics of the Studies Included in the Meta-analysis.

Source	Age at baseline	Boys, %	Follow-up, year	No. of Individuals/events	Exposure categories	Exposure assessment	Outcome and its assessment	Adjusted variables	Main results
Reilly, the ALSPC, 2005, UK	4 months to 5 years	50.7	~5	7,758/671	Quartiles of night sleep duration (hour/day): <10.5; 10.5–10.9; 11–11.9; ≥12.0.	Sleep duration in children aged 30 months was reported by parents.	Obesity: defined as BMI ≥ its 95^th^ percentile.	Maternal education, smoking during pregnancy, age of mother at delivery, gender, birth weight, parity, season of birth, gestational age, no of fetuses, infant feeding, parent obesity, no of siblings, energy intake at 3 years, ethnicity, television watching, time in car per day, and dietary patterns.	Obesity at age 7 [OR (95% CI)]:Q1: 1.45(1.10, 1.89);Q2: 1.35(1.02, 1.79);Q3: 1.04(0.76, 1.42);Q4: 1.00(Referent).
Lumeng, the NICHD-SECCYD study, 2007, USA	9 years	50.0	~3	785/139	Sleep time (hour/day) Sleep time in tertiles	Sleep duration was obtained by maternal report on CSHQ in the 3^rd^ and 6^th^ grades.	Obesity: defined as a BMI of ≥95^th^ percentile.	Gender, race, maternal education, change in sleep duration between 3^rd^ and 6^th^ grades, and the child’s weight status in the 3^rd^ grade.	Obesity in 6^th^ grade [OR (95% CI)]: 0.60(0.36, 0.99).T1: 3.48(1.09, 11.12);T2: 1.00(Referent);T3: 0.75(0.25, 2.30).
Snell, the PSID-CDS study, 2007, USA	3–12 years	50.0	~5	2,281/821	Sleep time(hour/day);Sleep time (hour/day):< 8; 8–8.9; 9–9.9; 10–10.9; ≥11.	The PSID-CDS time diaries were used to measure sleep time.	Standardized BMI by child’s age, gender and adjusted for skew. Overweight: following international guidelines (Cole, 2000).	BMI/overweight at time 1, gender, child’s age at time 1, child’s age at time 2, family income, average parental education in years, and race.	Subsequent age- and gender- standardized BMI (*β*±SE): −0.115±0.036.0.108±0.165;0.033±0.109;0(Referent);−0.164±0.086;−0.257±0.122.Overweight (*β*±SE):−0.053±0.017.−0.009±0.073;0.014±0.054;0(Referent);−0.070±0.044;−0.171±0.063.
Taveras, the Project Viva, 2008, USA	6 months to 2 years	50.0	1.75	915/82	Sleep duration (hour/day): <12;≥12.	Sleep duration at children’s age 6 months, 1 year and 2 years were reported by the mothers and weighted to get the average sleep time (hours/day).	BMI z-score; The sum of SS and TR skinfold thicknesses; Obesity: a BMI of ≥95^th^ age- and gender-specific percentile.	Maternal education, income, pre-pregnancy BMI, marital status, prenatal smoking history, breast feeding duration and child’s race/ethnicity, birth weight, 6-month weight-for-length z-score, daily television viewing, and daily active play.	Measures at end of follow-up:BMI z-score [*β*(95% CI)]:0.16(0.02, 0.29);0(Referent).Sum of SS and TR skinfold thicknesses:0.79(0.18, 1.40);0(Referent).Obesity[OR (95% CI)]:2.04 (1.07, 3.91);1.00(Referent).
Touchette, the QLSCD, 2008, Canada	29 months	46.8	~3.75	1,138/159	Sleep duration patterns: short persistent; short increasing; 10-hour persistent; 11-hour persistent.	Sleep duration was reported at 2.5, 3.5, 4, 5, and 6 years based on the last month by an open question from the sleep-administered questionnaire for mother.	Overweight and obesity were defined according to international standard definitions taking gender and age into account.	Birth weight, prematurity, low birth weight, gender of the child, maternal smoking during pregnancy, weight at 5 months, low parental education, modified family structure, late cereal introduction, not breast-fed, immigrant mother, naptime at 2.5 years, watching television at 6 years, playing video games at 6 years, doing physical activities, overeating at 6 years, snacking at 6 years, eating sweets at 6 years, snoring at 6 years, and low income status at 6 years.	Overweight/obesity at age 6 [OR (95% CI)]:short persistent:4.2(1.6, 11.1);short increasing:2.4(0.9, 6.4);10-hour persistent:1.8(1.1, 2.9);11-hour persistent:1.0 (Referent).
Bell, the PSID-CDS study,2010, USA	0–4 years	52	~5	822/271	Age-specific percentile of sleep duration: <25^th^ ; ≥25^th^.	The CDS questionnaire	Overweight: BMI>85^th^ and <95^th^ percentile. Obesity: BMI of ≥95^th^ percentile.	Age, gender, birth weight, father present, hours per day of television viewing, birth order, urban residence, child BMI z-score at baseline, race, family income, maternal education, parents’ BMI in 1999, and physical activity.	Overweight/obesity at end of follow-up [OR (95% CI)]:1.80(1.16, 2.80);1.00(Referent).
	5–13 years	50	~5	1,108/399	Age-specific percentile of sleep duration: <25^th^ ; ≥25^th^.	The CDS questionnaire	Overweight: BMI>85^th^ and <95^th^ percentile. Obesity: BMI of ≥95^th^ percentile.	Age, gender, birth weight, father present, hours per day of television viewing, birth order, urban residence, child BMI z-score at baseline, race, family income, maternal education, parents’ BMI in 1999, and physical activity.	Overweight/obesity at end of follow-up [OR (95% CI)]:1.21(0.85, 1.73);1.00(Referent)
Calamaro, the ADD health study, 2010, USA	16 years	50.3	~2	13,568/1,455	Duration of night sleep (hour/day): <6; 6~; 8~; 11-<14.	Adolescents were asked about the sleep duration during the in-home interview in both waves I and II.	Obesity, defined as BMI> 95^th^ age-specific percentile	Obesity at Wave I, age, gender, race, and parental income.	Obesity at wave II [OR (95% CI)]:1.41(0.87, 2.37);0.86(0.65, 1.14);1.00(Referent);1.15(0.56, 2.33).
Diethelm, the DONALD Study, 2010, Germany	~2 years	50.3	~5	481/NA	Sleep duration (hour/day): Consistently short; Inconsistent; Consistently long.	Sleep duration in children was reported by parents.	BMI gain; PBF gain; FFMI gain.	Gender, birth year, birth weight (<3000g) and rapid weight gain (0–18months).	Gains in outcomes of interest from age 2 to 7 (*β*±SE):BMI:0.12±0.11;−0.03±0.11; 0(Referent).FMI:0.15±0.07;−0.03±0.07; 0(Referent).FFMI:0.02±0.07;0.04±0.07; 0(Referent).
Carter, the FLAME study, 2011, New Zealand	3~5 years	56.0	~3	244/60	Sleep duration (hour/day)	Sleep duration were measured with Mini-Mitter omnidirectional Actical accelerometers attached by belts to the waist for five consecutive days, including two weekend days.	BMI; FMI; FFMI; Overweight (BMI≥85^th^ percentile).	Gender, maternal education, income, BMI, smoking during pregnancy, and income and child’s birth weight, ethnicity, physical activity, television viewing, non-core food intake, fruit and vegetable intake, and outcome of interest at baseline.	BMI at age 7:−0.39(−0.72, −0.06).FMI at age 7:−0.48(−0.86, −0.10).FFMI at age 7;−0.11(−0.29, 0.07).Overweight at age 7:0.44 (0.29, 0.67).
Hiscock, the LSAC, 2011, Australia	0–1 years	51.0	2	3,857/NA	Sleep duration (minute/day)	Sleep duration was measured using time-use diary reported by parents after each interview at wave 1 and 2.	BMI z-score: BMI was transferred to BMI z-score using the 2000 CDC growth reference.	Wave 1 gender and weight-for-age z-score.	BMI z-score at wave 2 [*β*(95%CI)]:−0.0002(−0.0005, 0.0001).
	4–5 years	51.7	2	3,844/NA	Sleep duration (minute/day)	Sleep duration was measured using time-use diary reported by parents after each interview at wave 1 and 2.	BMI z-score: BMI was transferred to BMI z-score using the 2000 CDC growth reference.	Wave 1 gender and BMI z-score.	BMI z-score at wave 2 [*β* (95%CI)]:−0.0002 (−0.0005, 0.0001).
Seeger, the QLSCD, 2011, Canada	10 years	50.1	3	1,916/194	TIB at 10 (continuous); TIB trajectory: Short sleepers; 10.5-hour sleepers; 11-hour sleepers.	Using open-ended questions, mothers were asked about their preadolescents’ bedtimes and waking times on weekdays at 10, 11, 12 and 13 years of age. Sleep duration was estimated as the TIB between bedtime and waking time.	BMI; The international definitions of overweight and obesity for preadolescents were based on BMI curves as a function of gender and age, as defined by Cole *et al.*	Gender, maternal immigrant status, familial income, birth weight, maternal and paternal educational levels, pubertal status, time spent watching television, and frequency of physical activity.	BMI at age 13 [*β*(95% CI)]:−0.71(−1.28, −0.14).1.24(0.38, 2.09);0.62(−0.01, 1.26);1.00 (Referent).Overweight at 13 [OR(95%CI)]:1.51(1.28, 1.76);1.99(1.67, 2.37);1.56(1.22, 1.99);1.00(Referent).Obesity [OR(95%CI)]:2.07(1.51, 2.84);2.23(2.18, 2.27);1.26(1.23, 1.29);1.00(Referent).
Silva, the TuCASA study, 2011, USA	6–12 years	51.0	4.8	304/106	Sleep duration (hour/night): ≥9; 7.5–9; <7.5.	Parents were asked to complete SHQs that inquired their children’s sleep history and sleep characteristics.	BMI; Overweight/obesity: BMI ≥85^th^ percentile; Obesity: BMI ≥95^th^ percentiles.	Ethnicity, baseline BMI, anxious/depressed, and learning problems, and age, SDB, and caffeine use at follow-up.	BMI gains [*β*(95% CI)]:0(Referent);1.06(−0.20, 2.10);1.70(0.40, 3.10).Overweight [OR(95%CI)]:1.0(Referent);1.5(0.69, 3.15);2.2(0.95, 5.09).Obesity [OR(95%CI)]:1.0(Referent);2.0(0.73, 5.64);3.3(1.09, 9.66).
Araújo, the EPITeen cohort study, 2012, Portugal	13 years	46.5	~4	1,171/NA	Sleep duration (hour/day)	Sleep duration was estimated by self-reported bedtimes and wake-up times at 17 years.	Change in BMI z-score from age 13 to 17; Change in PBF from age 13 to 17.	Parental education, KIDMED index, BMI z-score at age 13y, and PBF at age 13y.	*β*(95% CI) of change in BMI z-scores:Girls: 0.039(−0.006, 0.084)Boys: 0.010(−0.044, 0.065)*β*(95% CI) of change in PBF:Girls: 0.039(−0.139, 0.820)Boys: −0.334(−0.814, 0.146)
Klingenberg, the SKOT cohort study, 2012, Denmark	9 months	47.6	2.25	311/NA*	Sleep duration (hour/day)	Sleep duration including daytime napping from questionnaire data (TSD-Q) was based on parental report of time at age 9 months.	BMI z-score; SSF; PBF; FM.	Birth weight, gestational age, duration of breastfeeding, maternal smoking during pregnancy, maternal BMI at 9 months of examination, household income at time of investigation, and educational levels of both parents at time of investigation.	Adiposity at age 3 [*β*(95% CI)]: BMI z-score:−0.008(−0.13, 0.12).SSF: 0.289(−0.16, 0.73).PBF: −0.001(−0.003, 0.001).FM: −0.140(−0.35, 0.073).
	18 months	47.6	1.5	311/NA*	Sleep duration (hour/day)	Sleep duration including daytime napping from questionnaire data (TSD-Q) was based on parental report of time at age 18 months.	BMI z-score; SSF; PBF; FM.	Birth weight, gestational age, duration of breastfeeding, maternal smoking during pregnancy and maternal BMI at 9 months of examination, household income at time of investigation, and educational levels of both parents at time of investigation.	Adiposity at age 3 [*β*(95% CI)]:BMI z-score:−0.010(−0.07, 0.05).SSF: −0.039(−0.24, 0.16).PBF: 0.00005(−0.001, 0.001).FM: 0.024(−0.05, 0.09).
Lee, the Obesity and Metabolic Disorders Cohort in Childhood study, 2012, Korea	7 years	47.4	2	474/NA	Sleep duration (hours/day): <8.5; 8.5–9.5; ≥9.5.	Children and their parents were asked to fill out the questionnaires together about the sleep duration.	BMI gains during 2 year follow-up.	Age, gender, sexual maturation at 6^th^ year follow-up, baseline BMI, exercise, weekly screen time per an hour, household income, maternal BMI, paternal BMI, maternal education, paternal education, maternal job, family structure, energy intake, % of energy intake from fat, meal skipping during a week, and snacking status.	BMI gains[*β*(95% CI)]:0(Referent);−0.192(−0.543, 0.159);−0.463(−0.871, −0.054).
	10 years	49.3	2	1,030/NA	Sleep duration (hour/day): <8; 8–9; ≥9.	Children and their parents were asked to fill out the questionnaires together about the sleep duration.	BMI gains during 2 year follow-up.	Age, gender, sexual maturation at 6^th^ year follow-up, baseline BMI exercise, weekly screen time per an hour, household income, maternal BMI, paternal BMI, maternal education, paternal education, maternal job, family structure, energy intake, % of energy intake from fat, meal skipping during a week, and snacking status.	BMI gains[*β*(95% CI)]:0(Referent);−0.079(−0.321, 0.162);−0.024(−0.236, 0.285).
Lytle_male, The IDEA study and the ECHO study, 2012, USA	14.7 years	100	2	352/NA	Sleep duration (hour/day)	Total sleep duration was assessed via self-report.	BMI gains; PBF gains.	Race, grade, parental education, school lunch, puberty, study, screen time/sedentary behavior, depression, activity, and energy intake.	Gain in Adiposity(*β*±SE):BMI: −0.091±0.110.PBF: 0.105±0.306.
Lytle_female, The IDEA study and the ECHO study, 2012, USA	14.7 years	0	2	371/NA	Sleep duration (hour/day)	Total sleep duration was assessed via self-report.	BMI gains; PBF gains.	Race, grade, parental education, school lunch, puberty, study, screen time/sedentary behavior, depression, activity, and energy intake.	Gain in Adiposity(*β*±SE):BMI: 0.030±0.094.PBF: 0.259±0.226.
O’Dea, 2012, Australia	7–12 years	50.8	4	939/NA	Sleep duration during 4 years of follow-up: Consistently low; Intermediate; Consistently high.	Children and their mother completed an annual questionnaire (telephone interview) to assess usual amount of sleep.	BMI gains.	Age, birth weight, age of mother, maternal BMI, SES, mother smokes, father’s education, ethnicity, and physical activity.	The difference in BMI gains between children with consistently low and high sleep times was 1.00±0.45 kg/m^2^.
Storfer-isser, the CCSHS, 2012, USA	8–11 years	100	~8	157/NA	Sleep duration (hour/day)	Sleep duration was reported by the parents (ages 8–15) or the adolescent (ages 16–19).	Gender- and age-adjusted BMI z-score.	Age, race, low birth weight, low SES, and BMI z-score at baseline.	Subsequent BMI z-score (*β*±SE):Age 12–15:−0.08±0.08.Age 16–19:−0.06±0.08.
		0	~8	156/NA	Sleep duration (hour/day)	Sleep duration was reported by the parents (ages 8—15) or the adolescent (ages 16–19).	Gender- and age-adjusted BMI z-score.	Age, race, low birth weight, low SES, and BMI z-score at baseline.	Subsequent BMI z-score (*β*±SE):Age 12–15:0.01±0.07.Age 16–19:0.03±0.07.
Magee, the LSAC, 2013, Australia	Aged 0–1 years and 4–5 years	51.1	10	1,079/NA	Sleep duration (hour/day)	In waves 1, 2, and 3, sleep duration was assessed via time use diaries completed by one of the child’s parents.	BMI	Weekly household income, breastfed or not, the age the child stopped being breastfed completely, birth weight, mother/father education, and gender.	Shorter sleep duration are primarily associated with BMI in children with early onset obesity(a subgroup): Sleep duration at age 6 to 7 was inversely associated with BMI at age 8 to 9 years (*β* = −0.68, *P* = 0.017); and sleep duration at age 8 to 9 was inversely associated with BMI at age 10 to 11 years (*β* = −1.21, *P* = 0.003).
Mitchell, 2013, USA	14 years	50	3.4 (3–4)	1,390/NA	Sleep duration (hour/day)	Typical duration of sleep on a school night and on a weekend night was self-reported by the participants.	BMI gains	Gender, race, maternal education, MVPA, and screen time.	50^th^ percentile of BMI [*β*(95% CI)]:−0.15(−0.24,−0.06).
Hjorth, the OPUS school meal study, 2014, Denmark	8–11 years	51.7	0.55	723/NA	Sleep duration (hour/day)	The parents and children were instructed to keep logs for bedtime (“lights off” and trying to sleep) and waking time (“lights on”) during the week in which the monitor was worn.	Change in WC	Baseline age, gender, pubertal status, gender-pubertal status interaction, days of follow up and baseline WC.	*β*(95% CI):−0.10(−0.67, 0.46).
Magee, the LSAC, 2014, Australia	4–5 years	52.4	4	2,984/NA	Sleep duration (hour/day)	Sleep duration was reported by parents by self-report questionnaires and time use diaries.	BMI	Gender, sleep problems, household income, maternal education, and maternal weight status.	Short sleep duration at age 4 to 5 years was significantly associated with higher BMI at age 8 to 9 years (*β* = −0.07, *P* = 0.044), which was slightly attenuated by television viewing at age 6 (*β* = −0.06, *P* = 0.076).
Michels, Belgian longitudinal ChiBS study, 2014, Belgium.	6–12 years	52	~2	193/NA	Sleep duration (hour/day)	Sleep duration in children was reported by parents using a sleep diary	BMI z-score gains; PBF gains;WC gains.	Age, gender, parental education, physical activity and reported snacking frequency.	Adiposity evolution over 2 years: BMI z-score[*β* (p-value)]: −0.381(0.030);PBF [*β*(p-value)]: −2.348 (0.002); WC[*β*(p-value)]: −1.666(0.016).
Scharf, the ECLS-B, 2014, USA	9 months	50.9	5	8,950/NA	Sleep time (hour/day)	The primary care giver (most often the mother) completed a computer-assisted interview at home by trained assessors.	Increases in BMI z-score.	Gender, race/ethnicity, socioeconomic status, and television viewing.	Increases in BMI z-score (beta±SE):−0.0287±0.0127
Taveras, the Project Viva, 2014, USA	6 months to 2 years	49.7	7.2	1,046/116*	Curtailed sleep (hour/day): <12; ≥12.	Mothers reported their baby’s sleep duration in a usual 24-hour period at 6 months and 1 year old.	BMI z-score; FMI; WC; Obesity: BMI for age and gender ≥95^th^ percentiles.	Child’s age and gender, maternal age, education, and parity, household income and child’s race and television viewing at mid-childhood.	BMI z-score[*β* (95%CI)]:0.15(0.02, 0.28);0(Referent).FMI[*β* (95%CI)]:0.19(−0.05, 0.43);0(Referent).WC[*β* (95%CI)]:1.02(0.03, 2.01);0(Referent).Obesity[OR (95% CI)]:1.36(0.84, 2.21);1.00(Referent).
	3–4 years	49.7	4.4	1,046/116*	Curtailed sleep (hour/day): <10; 10- < 11; ≥11.	Parents reported their children’s sleep duration in a usual 24-hour period on average weekday and weekend day in the past month.	BMI z-score; FMI; WC; Obesity: BMI for age and gender ≥95^th^ percentiles.	Child’s age and gender, maternal age, education, and parity, household income and child’s race and television viewing at mid-childhood.	BMI z-score[*β* (95%CI)]:0.20(−0.01, 0.40);0.07(−0.07, 0.20);0(Referent).FMI[*β* (95%CI)]:0.15(−0.25, 0.54);0.07(−0.17, 0.32);0(Referent).WC[*β* (95%CI)]:0.99(−0.64, 2.62);0.46(−0.57, 1.48);0(Referent).Obesity[OR (95% CI)]:1.27(0.64, 2.52);0.98(0.58, 1.66);1.00(Referent).
	5–7 years	49.7	1.7	1,046/116*	Curtailed sleep (hour/day): <9; 9-<10; ≥10.	Mothers reported their children’s sleep duration in a usual 24-hour period on average weekday and weekend day in the past month.	BMI z-score; FMI; WC; Obesity: BMI for age and gender ≥95^th^ percentiles.	Child’s age and gender, maternal age, education, and parity, household income and child’s race and television viewing at mid-childhood.	BMI z-score[*β* (95%CI)]:0.24(−0.08, 0.56);0.01(−0.15, 0.18);0(Referent).FMI[*β* (95%CI)]:0.33(−0.32, 0.98);0.05(−0.26, 0.36);0(Referent).WC[*β* (95%CI)]:1.92(−0.72, 4.56);0.36(−0.94, 1.67);0(Referent).Obesity[OR (95% CI)]:2.12(0.90, 4.98);1.09(0.60, 2.00);1.00(Referent).

ADD: the National Longitudinal Study of Adolescent Health; ALSPAC : Avon Longitudinal Study of Parents And Children; BMI: body mass index; CCSHS: Cleveland Children’s Sleep and Health Study; CDC: Center of Disease Control; CDS: Child Development Supplement; ChiBS: Children’s Body composition and Stress; CI: confidence interval; CSHQ: Children’s Sleep Habits Questionnaire; DONALD: Dortmund Nutritional and Anthropometric Longitudinally Designed; ECHO: Etiology of ChildHood Obesity; ECLS-B: the Early Childhood Longitudinal Study-Birth Cohort; EPITeen: Epidemiological Investigation of Teenagers Health in Porto; FFMI: fat free mass index; FM: fat mass; FMI: fat mass index; FLAME: Family Lifestyle, Activity, Movement and Eating study; IDEA: International Day for the Evaluation of Abdominal Obesity; IOTF: International Obesity Task Force; KIDMED: Mediterranean Diet Quality Index in Children and Adolescents; LSAC: Longitudinal Study of Australian Children; MVPA: moderate-to-vigorous physical activity; NA: not applicable; NICHD-SECCYD: National Institute of Child Health and Human Development Study of Early Child Care and Youth Development; OPUS: Optimal well-being, development and health for Danish children through a healthy New Nordic Diet; OR: odds ratio; PBF: Percentage body fat; PIAMA: Prevention and Incidence of Asthma and Mite Allergy study; PSID: the Panel Survey of Income Dynamics; QLSCD: Quebec Longitudinal Study of Child Development; SDB: sleep disordered breathing; SES: social economic status; SHQ: sleep habits questionnaire; SKOT: Småbørns Kost Og Trivsel (in Danish, which means “Toddlers Diet And Welfare” in English); SS: subscapular; SSF: sum of the skin-fold; TIB: time in bed; TR: triceps; TuCASA: Tucson Children’s Assessment of Sleep Apnea Study; UK: the United Kingdom; USA: the United States of America; WC: waist circumference.

^*^The numbers of participants and events, i.e. 1,046/116, are for the whole study across three age groups.

**Table 2:** Stratified analyses of the association between sleep duration and risk of overweight/obesity or annual BMI gain.

Outcome	Sleep duration	Potential modifiers		No of cohorts	No of participants/events	Heterogeneity test	OR/*β* (95% CI)	*P*for interaction
Overweight/Obesity	lowest *vs*. highest	Baseline age, year	<3	5	11,679/1,299	*I*^2^ = 28.1%,*P* = 0.23	1.68 (1.30, 2.16)	0.61
			3~<5	1	1,046/116	NA	1.27 (0.64, 2.52)	
			≥5	4	4,374/815	*I*^2^ = 28.1%,*P* = 0.23	1.90 (1.24, 2.91)	
		Follow-up period, year	<3.4	3	3,877/392	*I*^2^ = 51.0%,*P* = 0.13	1.95 (1.44, 2.65)	0.34
			≥3.4	7	12,176/1722	*I*^2^ = 35.3%,*P* = 0.16	1.62 (1.26, 2.09)	
		Study location	USA	7	4,195/974	*I*^2^ = 0.0%,*P* = 0.43	1.52 (1.24, 1.86)	0.29
			Non-USA	3	10,812/1024	*I*^2^ = 82.3%, *P *< 0.01	2.03 (1.37,3.01)	
Overweight/Obesity	↑ 1 hour/day	Baseline age, year	<3	1	7,758/671	NA	0.86 (0.78, 0.94)	0.74
			3 ~ <5	2	1,290/176	*I*^2^ = 82.3%,*P* < 0.01	0.65 (0.31, 1.34)	
			≥5	6	20,815/2,913	*I*^2^ = 82.3%,*P* < 0.01	0.79 (0.67, 0.92)	
		Follow-up period, year	<3.4	4	11,389/1,714	*I*^2^ = 87.6%, *P *< 0.01	0.64 (0.44, 0.93)	0.20
			≥3.4	5	17,559/1,964	*I*^2^ = 63.1%, *P *< 0.01	0.89 (0.80, 0.98)	
		Study location	USA	6	17,984/2,637	*I*^2^ = 89.8%, *P *< 0.01	0.91 (0.83, 0.998)	0.13
			Non-USA	3	9,918/925	*I*^2^ = 47.5%,*P* = 0.09	0.58 (0.36, 0.95)	
Annual BMI gain	lowest *vs*. highest	Baseline age, year	<3	1	481/NA	NA	0.02 (−0.02, 0.07)	0.51
			3~<5	0	—	—	—	
			≥5	4	2,747/NA	*I*^2^ = 47.5%,*P* = 0.09	0.19 (0.13, 0.35)	
		Follow-up period, year	<3.4	2	1,504/NA	*I*^2^ = 68.1%,*P* = 0.08	0.11 (−0.11, 0.32)	0.72
			≥3.4	3	1,724/NA	*I*^2^ = 77.3%,*P* = 0.01	0.18 (−0.04, 0.40)	
		Study location	USA	1	304/NA	NA	0.35 (0.07, 0.64)	0.26
			Non-USA	4	2,924/NA	*I*^2^ = 60.2%,*P* = 0.06	0.09 (−0.02, 0.19)	
Annual BMI gain	↑ 1 hour/day	Baseline age, year	<3	0	—	—	—	—
			3 ~ <5	0	—	—	—	
			≥5	6	3,921/NA	*I*^2^ = 42.7%,*P* = 0.21	−0.05 (−0.09, −0.01)	
		Follow-up period, year	<3.4	4	2,227/NA	*I*^2^ = 33.6%,*P* = 0.21	−0.04 (−0.09, 0.02)	0.46
			≥3.4	2	1,694/NA	*I*^2^ = 71.6%,*P* = 0.06	−0.08 (−0.15, 0.00)	
		Study location	USA	4	2,417/NA	*I*^2^ = 44.5%,*P* = 0.14	−0.05 (−0.10, −0.00)	0.97
			Non-USA	2	1,504/NA	*I*^2^ = 69.5%,*P* = 0.07	−0.06 (−0.15, 0.04)	

BMI: body mass index; CI: confidence interval; NA: not applicable; OR: odds ratio; USA: the United States of America.

**Table 3:** The association of sleep duration with secondary outcomes

Outcome	Exposure	No. of cohorts	No of participants	*β* (95% CI)	Heterogeneity test
BMI, kg/m^2^					
End of follow-up	Lowest *vs*. highest	1	1,916	1.24 (0.39, 2.09)	—
End of follow-up	↑ 1 hour/day	5	5,814	**−0.51 (−0.88, −0.14)**	*I*^2^ = 83.8%; *P *< 0.01
BMI (standardized), kg/m^2^					
Annual gain	↑ 1 hour/day	3	3,438	−0.02 (−0.06, 0.02)	*I*^2^ = 82.8%; *P *< 0.01
End of follow-up	Lowest *vs*. highest	4	1,961	**0.17 (0.08, 0.25)**	*I*^2^ = 49.6%; *P* = 0.03
End of follow-up	↑ 1 hour/day	11	11,652	**−0.04 (−0.07, −0.02)**	*I*^2^ = 0.0%; *P* = 0.95
WC, cm		2			
Annual gain	↑ 1 hour/day	2	916	−0.40 (−0.06, 0.02)	*I*^2^ = 61.5%; *P* = 0.11
End of follow-up	Lowest *vs*. highest	3	1,046	**1.10 (0.29, 1.90)**	*I*^2^ = 0.0%; *P* = 0.81
End of follow-up	↑ 1 hour/day	2	1,046	**−0.54 (−1.06, −0.01)**	*I*^2^ = 0.0%; *P* = 0.76
PBF, %					
Annual gain	↑ 1 hour/day	4	2,087	−0.06 (−0.30, 0.18)	*I*^2^ = 73.5%; *P* = 0.01
End of follow-up	↑ 1 hour/day	2	311	−0.00 (−0.00, 0.00)	*I*^2^ = 0.0%; *P* = 0.35
FFMI					
Annual gain	Lowest *vs*. highest	1	481	0.00 (−0.02, 0.03)	—
End of follow-up	↑ 1 hour/day	1	244	−0.11 (−0.29, 0.07)	—
FM					
End of follow-up	↑ 1 hour/day	2	311	−0.03 (−0.17, 0.12)	*I*^2^ = 51.8%; *P* = 0.15
FMI					
Annual gain	Lowest *vs*. highest	1	481	**0.03 (0.003,0.06)**	—
End of follow-up	Lowest *vs*. highest	3	1,046	−0.16 (−0.34, 0.03)	*I*^2^ = 47.7%; *P* = 0.15
End of follow-up	↑ 1 hour/day	3	1,290	0.19 (−0.00, 0.39)*	*I*^2^ = 0.0%; *P* = 0.90
SSF					
End of follow-up	↑ 1 hour/day	2	311	0.06 (−0.23, 0.36)	*I*^2^ = 42.4%; *P* = 0.19

BMI: body mass index; FFMI: fat free mass index; FM: fat mass; FMI: fat mass index; PBF: Percentage body fat; SSF: sum of the skin-fold; WC: waist circumference.

^*^P = 0.053
