# Supplementary material for: Habitual Sleep Duration and Risk of Childhood Obesity: Systematic Review and Dose-response Meta-analysis of Prospective Cohort Studies
Source: Sci Rep. 2015 Nov 5;5:16160. doi: 10.1038/srep16160 (PMC4633618; doi:10.1038/srep16160)
Supplement: Supplementary Information [file srep16160-s1.pdf]

Habitual Sleep Duration and Risk of Childhood Obesity: Systematic Review and Dose-response Meta-analysis of Prospective Cohort Studies

Huijuan Ruan[1†], Pengcheng Xun[2†], Wei Cai[1,3,4], Ka He[2*] and Qingya Tang[1*]

[1]Department of Clinical Nutrition, Xin Hua Hospital, School of Medicine, Shanghai Jiao Tong University, Shanghai, China;

[2]Department of Epidemiology and Biostatistics, School of Public Health-Bloomington, Indiana University, Bloomington, IN, USA;

[3]Shanghai Key Laboratory of Pediatric Gastroenterology and Nutrition, Shanghai, China; Shanghai Institute of Pediatric Research, Shanghai, China;

[4]Department of Pediatric Surgery, Xin Hua Hospital, School of Medicine, Shanghai Jiao Tong University, Shanghai, China.

[†]These authors contribute equally to this work.

[*]**Corresponding authors:**

Dr. Qingya Tang; Address: No. 1665, Kongjiang Rd, Shanghai, China; Phone: +86-21-25078670; Fax: +86-21-25078972; E-mail: tangqingya@163.com

Dr. Ka He; Address: Department of Epidemiology and Biostatistics, School of Public Health-Bloomington, Indiana University, 1025 E. Seventh Street, C034, Bloomington, Indiana 47405, USA; Phone: 812-856-2260; Fax: 812-855-7345; E-mail: kahe@indiana.edu

Supplemental Table Influence of single study on the association between sleep duration and risk of overweight/obesity or annual BMI gain

**Outcome**	**Comparison**	**Cohort omitted**	**OR (95% CI)**
Overweight/obesity	Lowest *vs*. highest	None	**1.76(1.39, 2.23)**
		Reilly, 2005	1.83 (1.42, 2.34)
		Taveras, 2008	1.74 (1.35, 2.24)
		Touchette, 2008	1.69 (1.33, 2.15)
		Bell_younger, 2010	1.76 (1.36, 2.29)
		Bell_older, 2010	1.86 (1.49, 2.33)
		Seeger, 2011	1.59 (1.30, 1.93)
		Silva, 2011	1.72 (1.35, 2.19)
		Teveras_0.5-2 years, 2014	1.82 (1.42, 2.32)
		Teveras_3-4 years, 2014	1.81 (1.42, 2.30)
		Teveras_5-7 years, 2014	1.75 (1.36, 2.24)
	↑ 1 hour/day	None	**0.79 (0.70, 0.89)**
		Reilly, 2005	0.76 (0.65, 0.88)
		Lumeng, 2007	0.80 (0.71, 0.91)
		Snell, 2007	0.73 (0.61, 0.87)
		Calamaro, 2010	0.74 (0.63, 0.86)
		Seeger, 2011	0.83 (0.74, 0.93)
		Carter, 2011	0.85 (0.76, 0.94)
		Silva, 2011	0.80 (0.70, 0.91)
		Teveras_3-4 years, 2014	0.77 (0.68, 0.88)
		Teveras_5-7 years, 2014	0.79 (0.69, 0.90)
**Outcome**	**Comparison**	**Cohort omitted**	***β*(95% CI)**
Annual BMI gain	Lowest *vs*. highest	None	**0.13 (0.01, 0.25)**
		Diethelm, 2010	0.19 (0.03, 0.35)
		Silva, 2011	0.09 (-0.02, 0.19)
		Lee_1st grader, 2012	0.10 (-0.02, 0.23)
		Lee_4th grader, 2012	0.19 (0.01, 0.36)
		O'Dea, 2012	0.10 (-0.02, 0.22)
	↑ 1 hour/day	None	**-0.05 (-0.09, -0.01)**
		Silva, 2011	-0.04(-0.07, -0.01)
		Lee_1st grader, 2012	-0.04 (-0.08, -0.01)
		Lee_4th grader, 2012	-0.06 (-0.10, -0.02)
		Lytle_females, 2012	-0.06 (-0.10, -0.02)
		Lytle_males, 2012	-0.05 (-0.09, -0.01)
		Mitchell, 2013	-0.05 (-0.11, -0.00)

BMI: body mass index; CI: confidence interval; OR: odds ratio.
